# A compensatory relationship between working and long-term memory for emotional faces

**DOI:** 10.3389/fpsyg.2025.1716951

**Published:** 2026-01-09

**Authors:** Jongsoo Baek, Hangyeol Son, Dongjin Shin, Jae-Won Yang

**Affiliations:** 1 Department of Philosophy, College of Humanities, Kyung Hee University, Seoul, Republic of Korea; 2 Department of Psychology, The Catholic University of Korea, Bucheon-si, Gyeonggi-do, Republic of Korea

**Keywords:** arousal-biased competition, emotional bias, emotional memory, facial expressions, long-term memory, memory trade-off, resource allocation, working memory

## Abstract

**Introduction:**

Previous research has presented contradictory findings regarding emotional memory enhancement, often demonstrating an “angry face advantage” in working memory (WM) and a “happy face advantage” in long-term memory (LTM). However, the relationship between emotional memory enhancements in WM and LTM, particularly at the individual level, remains unclear. This study investigated the change in emotional memory for happy versus angry faces from immediate to delayed recognition and explored the individual-level correlation between WM and LTM performance.

**Methods:**

A memory experiment was conducted over two consecutive days. On the first day, participants completed a change-detection task to measure WM for angry and happy faces. Approximately 24 h later, a surprise LTM recognition task was administered to assess the memory of the facial identities encountered during the WM task. This study measured WM capacity and LTM sensitivity for both emotional faces, and a correlation analysis was performed between individual bias scores (difference in memory for happy versus angry faces) in WM and LTM tasks.

**Results:**

There was no significant difference in memory capacity in the group-level analysis of WM performance for happy and angry faces. In contrast, the delayed LTM test showed a robust happy face advantage, with significantly better recognition of faces initially encountered with happy expressions than with angry expressions. Most critically, the correlation analysis revealed a significant negative relationship between WM and LTM emotional biases, indicating that individuals with relatively better WM performance for one emotional valence subsequently exhibited a reduced LTM advantage for the same valence.

**Discussion:**

The negative correlation between WM and LTM emotional biases aligns with the arousal-biased competition model, suggesting that the cognitive resources allocated to maintaining threatening information in WM may come at the cost of elaborate encoding processes necessary for robust LTM consolidation. These results provide novel evidence for a compensatory processing mechanism wherein the cognitive system manages immediate and delayed memories of emotional faces in a complementary rather than cumulative fashion.

## 1 Introduction

Emotional facial expressions serve as significant social signals. Facial expressions facilitate the prediction of others’ emotional states and intentions, thereby allowing the adjustment of one’s own behavior (Adolphs, 2003). For example, an angry face rapidly signals potential threats, aggression, or conflict and the immediate need for a defensive response. In contrast, a smiling face signals affiliation, trust, safety, and social rewards, promoting approach and cooperation. Given these distinct adaptive values and their social significance, it is not surprising that emotionally salient faces influence how the cognitive system processes and retains information. Emotional memory literature has reported that emotional faces tend to be remembered better than neutral faces (D’Argembeau and Van der Linden, 2007; Hamann, 2001; Kensinger, 2009; Lee and Cho, 2019; Murty et al., 2010; Righi et al., 2012; Sergerie et al., 2007). However, the enhancement of emotional memory is a complex phenomenon that often appears contradictory. Previous studies have revealed that the memory advantage for different emotional expressions (angry versus happy) may vary depending on the memory system involved (working memory versus long-term memory) and the temporal scale of the memory test (immediate versus delayed) (D’Argembeau and Van der Linden, 2007; Jackson et al., 2009; Shimamura et al., 2006).

Numerous studies examining immediate memory or working memory (WM) for emotional faces have frequently demonstrated an “angry face advantage,” which refers to the superior processing and retention of threatening facial expressions in WM (Feldmann-Wüstefeld et al., 2011; Jackson et al., 2009, 2012). In a change-detection task, for instance, participants demonstrated a higher degree of precision in maintaining angry faces in the WM than happy or neutral faces; however, there was no significant difference in WM between happy and neutral faces, suggesting a specific enhancement for anger rather than an impairment for happiness (Jackson et al., 2009).

The angry face advantage is attributed to several cognitive mechanisms, including rapid attentional capture, enhanced encoding precision, and preferential maintenance of negative emotional information in WM. Angry faces are known to capture attention rapidly (Fox et al., 2000; Hansen and Hansen, 1988; Öhman et al., 2001). Electrophysiological studies have demonstrated that angry faces, even when task-irrelevant (Burra et al., 2016), elicit stronger and earlier neural responses of attention than happy faces, indicating a differential allocation of attention (Feldmann-Wüstefeld et al., 2011; Schupp et al., 2004). Heightened attention at an early stage could lead to greater precision during encoding, because stimuli that receive more attention are encoded more accurately (Jackson et al., 2014; Lee and Cho, 2019). Moreover, negative emotional information appeared to be preferentially sustained during WM maintenance (Jackson et al., 2009). When the encoding time and retention intervals are short, prioritized items effectively occupy limited WM slots or are represented at a higher resolution.

It is worth noting that not all studies examining WM for facial emotions have found the angry face advantage. Research has yielded mixed results, with some studies reporting no differential effects between happy and angry facial expressions on WM performance (Curby et al., 2019; Langeslag et al., 2009). These inconsistencies may reflect methodological differences, such as the tasks and stimuli used in the experiments or individual differences in traits, including anxiety and attentional control.

In contrast to the angry face advantage for WM, numerous studies have consistently reported a “happy face advantage” in long-term memory (LTM), where happy facial expressions are remembered more accurately than angry or neutral expressions. D’Argembeau and Van der Linden (2007) demonstrated that recognition performance for facial identity was better for faces initially displayed with a happy expression than for those with an angry one. Shimamura et al. (2006) found that happy expressions were remembered better than angry, fearful, and surprised expressions. This advantage persisted even when the faces were inverted to disrupt configural processing or when salient perceptual features, such as a broad grin, were controlled. These studies supported the idea that happy faces are more readily remembered in the LTM.

The contrasting patterns of emotional effects in WM and LTM present a counterintuitive memory trade-off effect that challenges the traditional multistore model of memory. The conventional model posits that enhanced WM representations result in better performance on LTM tasks (Atkinson and Shiffrin, 1968; Forsberg et al., 2020). From this perspective, enhanced WM for angry faces should translate into superior LTM. However, this prediction is contradicted by the positivity bias in LTM. The research cited above showed that emotional memory benefits are nuanced depending on the emotional valence and memory system. Therefore, to understand the dynamic interplay between memory systems for emotional faces and the time course of emotional information in cognitive processes, emotional memory enhancement for happy and angry faces in both WM and LTM should be compared within individuals. While most previous studies examined the emotional effects on either WM or LTM in isolation, few have systematically investigated the relationship between these effects within the same participants (but see Dolcos et al., 2013). This methodological gap limits our understanding of the potential individual-level relationships between immediate and delayed emotional memory processes. To determine the relationship between these conflicting emotional effects on memory - whether emotional effects on WM and LTM are independent phenomena that are common in most people or maintaining one emotional valence in WM come at the cost of effective long-term encoding for that same valence - it is necessary to investigate not only divergent group-level effects of emotional expressions on WM and LTM, but also the relationship between these effects at the individual level.

The primary purpose of the current study was to examine how emotional memory for angry versus happy faces changes from immediate to delayed recognition, and crucially, to explore the individual-level correlation between these two measures. To this end, we conducted a within-subjects experiment wherein participants performed a WM task for happy and angry faces, followed by a surprise LTM test after a 24-h delay. This design allowed us to measure both the immediate WM capacity for each type of emotional face and the LTM for the same faces after consolidation. By examining both group-level differences in these measures and their correlation across individuals, we clarified how the emotional valence effect shifts over time and whether WM and LTM advantages indeed trade-off. This approach yielded new insights into the dynamics of emotional memory and competition for cognitive resources.

## 2 Methods

The experiment was conducted over two consecutive days. On the first day, the participants engaged in a change-detection task to measure WM for angry and happy faces. On the second day, they performed a recognition task to evaluate LTM for the stimuli encountered during the WM task.

### 2.1 Participants

Fifty-one Korean undergraduate students participated in the experiment (26 males and 25 females; aged 18 to 28 years; *M_age_* = 22.37, *SD_age_* = 2.71) in exchange for a monetary compensation of $8 USD. The sample size was determined by a power analysis. Based on a meta-analysis reporting a median effect size of *r* = 0.43 in experimental psychology (Schäfer and Schwarz, 2019), we set a target effect size of *r* = 0.40 (medium-to-large effect size). To achieve a statistical power of 0.80 at an alpha level of 0.05 (two-tailed), a minimum of 46 participants was required. Considering the potential exclusion of individuals with high social anxiety, an additional 10% of participants were recruited, resulting in a total sample size of 51. Written informed consent was obtained from all the participants. This study was approved by the Institutional Review Board of Kyung Hee University.

### 2.2 Stimuli

**Figure 1:**
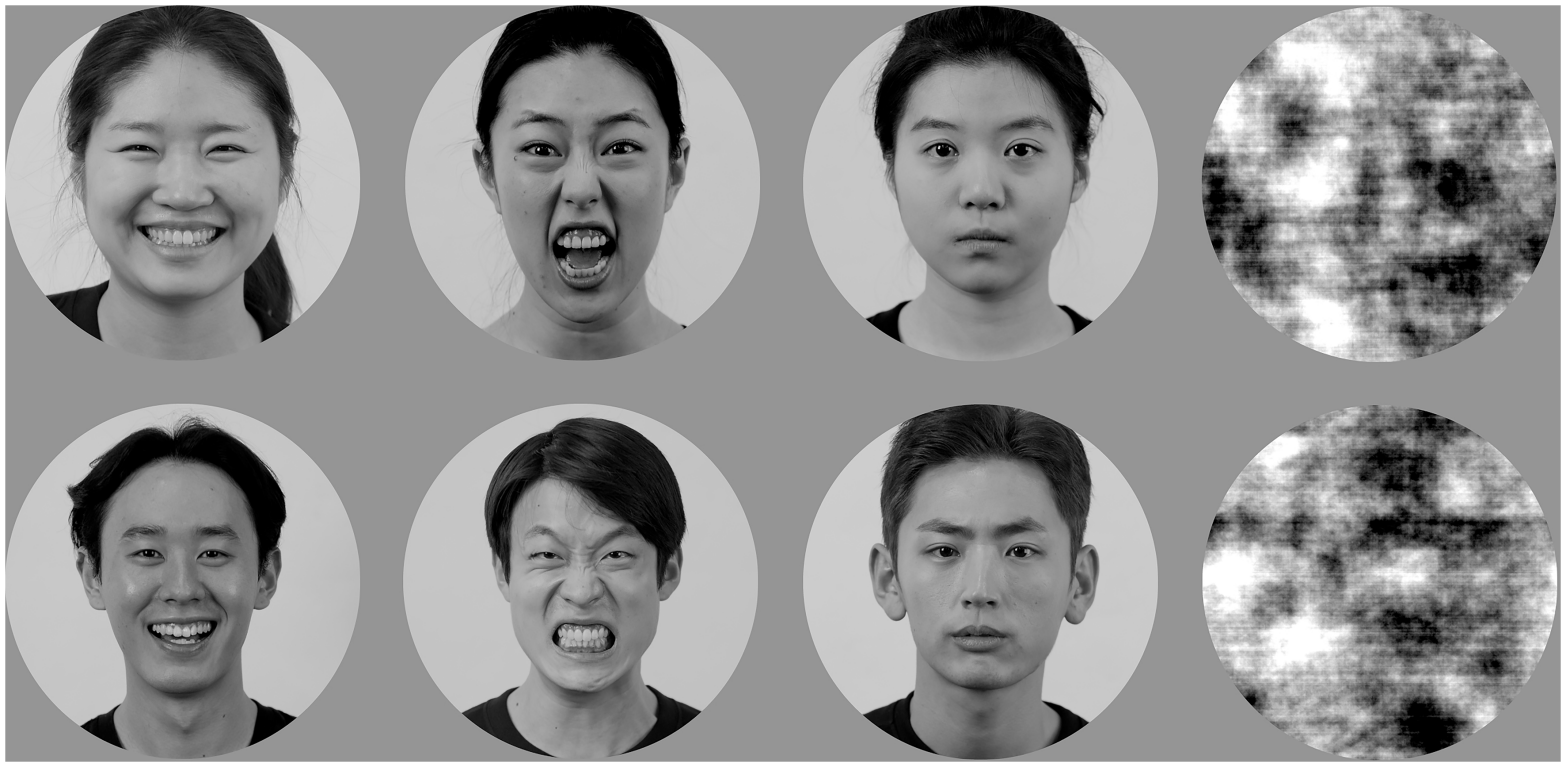
Examples of facial expression stimuli used in this study: positive (happy), negative (angry), and neutral expressions from left to right columns, and scrambled images in the rightmost column. Top row for female actors, and bottom row for male actors.

The stimuli consisted of facial pictures expressing various emotions drawn from the Yonsei Face Database (Chung et al., 2019). The pictures in this database can be considered prototypical exemplars of each emotion because the facial expressions in these pictures have been reported to be highly accurate (the percentage of correct classifications across the seven basic emotion categories was 99.02% for happiness and 86.71% for anger) and intense (5.34 for happiness and 5.37 for anger on a 7-point Likert scale). Of the 74 Korean actors in the database, each of whom had angry, happy, and neutral expressions, 72 (36 males and 36 females) were selected. The dimensions, alignment, and orientation of each face were standardized based on the position of the eyes and the tip of the nose. The images were then converted to grayscale and cropped into circular shapes. The brightness and contrast were standardized. Examples of these stimuli are shown in Figure 1.

For the WM task, the stimuli consisted of 48 facial pictures: 24 actors’ happy faces and another 24 actors’ angry faces, randomly selected for each participant before the experiment. The facial pictures of the remaining 24 actors were not used in the WM task. For each trial, we randomly selected one to four pictures of actors of the same gender expressing the same emotion from the participant’s pre-selected pool of 48 faces (24 happy + 24 angry) to generate a memory array. Each facial picture was expected to appear approximately 16.67 times within memory arrays and 6.67 times as a test stimulus during the WM task, ensuring balanced exposure frequency across stimuli. In trials with fewer than four memory loads, filler images were presented at the remaining stimulus locations. The fillers were generated by scrambling the Fourier phase of the original facial stimulus. Scrambled images preserve the low-level visual properties of the original image, while disrupting its recognition by maintaining the amplitude spectrum and randomizing the phase information (LoPresti et al., 2008).

For the LTM recognition task, the set of “old” items comprised all the facial identities presented with happy or angry expressions during the WM task. The test faces in the LTM task were always presented with neutral expressions, irrespective of their expressions during the WM task. Another 24 novel facial identities, also with neutral expressions, served as the “new” items for the recognition test.

### 2.3 Procedure

#### 2.3.1 Measures of social anxiety

Before the WM experiment, the participants completed the Social Interaction Anxiety Scale (SIAS) (Mattick and Clarke, 1998) to assess the level of distress or anxiety experienced during social interactions. They were instructed to indicate the degree to which each of the 20 statements described them on a 5-point Likert scale. A SIAS score of 37 was suggested as the clinical cut-off, which indicates probable social anxiety disorder (Peters, 2000).

#### 2.3.2 WM task

**Figure 2:**
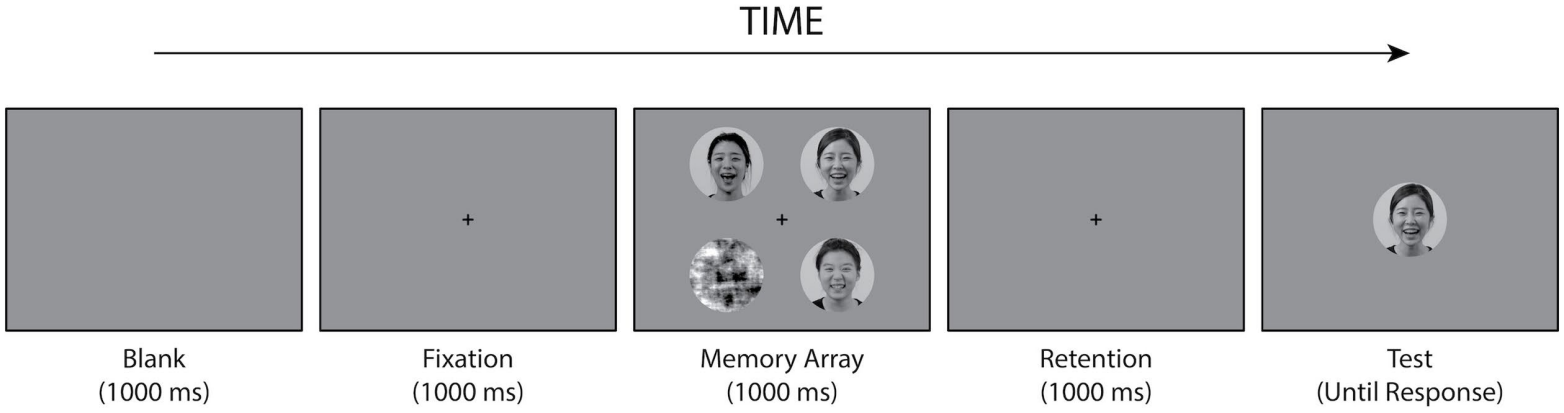
Experimental procedures in the WM task. In each trial, the participants were shown a memory array containing 1–4 facial pictures with the same emotional expressions (e.g., all happy faces in this example) for 1,000 ms. After a delay of 1,000 ms, a test face was presented until a response was given. The test face was either one of the faces in the memory array or a new facial picture. Participants were instructed to indicate whether the test stimulus was presented or not in the memory array.

The WM task was a change-detection task with a single probe similar to that described by Jackson et al. (2009). This phase primarily assessed the WM for facial identity. The experiment incorporated two within-subject independent variables: facial expression during encoding (faces displaying angry or happy emotional expressions) and WM load (the number of faces in the memory array). At the beginning of each trial, a fixation cross was presented for 1,000 ms at the center of the screen. Following the fixation, a memory array of 1–4 faces was simultaneously presented around the fixation point for 1,000 ms. For trials in which the number of memory loads was less than four, scrambled images were presented at the remaining stimulus locations. All the stimuli were presented on a gray background. The size of each stimulus was 4.56° of visual angle (diameter). Each stimulus was positioned at a diagonal distance of 4.83° from the central fixation, with one stimulus placed in each of the four visual quadrants. A retention interval of 1,000 ms was presented, following the memory array. A test face was then presented centrally until the participants responded. The participants were instructed to indicate whether the test stimulus was presented in the preceding memory array. The experiment employed a full factorial design across emotional valence (happy vs. angry), stimulus gender (male vs. female), and WM Load (1, 2, 3, and 4). Therefore, the number of trials was balanced between male and female stimuli and between happy and angry stimuli. All experimental conditions were randomly interleaved within each block. No feedback was provided. Examples of the stimuli and procedures used in each trial are shown in Figure 2. The participants performed a concurrent articulatory suppression task. Two letters appeared on the screen at the beginning of each block, and the participants were required to pronounce them continuously throughout the block. After completing each block, they were presented with either the same or different pairs of letters and had to determine whether the letters at the end matched those at the beginning. The experiment consisted of five blocks, each of which included 64 trials. There was a 30-s break between each block.

#### 2.3.3 LTM task

Approximately 24 h after the WM experiment, the participants returned to complete the LTM recognition task. They were not informed of the subsequent memory tests on Day 1. This phase aimed to assess the long-term recognition of facial identity and examine any biases related to the emotional expression of the faces encountered during the initial encoding phase on Day 1. In each trial, a single face was presented at the center of the screen. The stimuli included neutral faces of actors who were shown with either happy or angry emotions on day 1, as well as novel faces that had not been presented before. The participants were instructed to indicate whether each face was presented in the WM experiment on Day 1.

### 2.4 Apparatus

The experiment was controlled by a PC using the PsychoPy software (Peirce, 2007). The stimuli were presented on a 27” LED monitor with a resolution of 1,920 × 1,080 pixels and a refresh rate of 60 Hz and viewed from a distance of approximately 50 cm with a chinrest. The responses were recorded using a standard keyboard.

### 2.5 Data analysis

The data analysis for the WM experiment conducted on Day 1 was largely consistent with the established procedures delineated in previous studies (Jackson et al., 2009, 2014). The WM performance measures included the sensitivity index, *d’*, which indicates how well a participant recognizes the facial stimuli in the memory array. For each participant, we computed hit rates (proportion of “present” responses in trials in which a test stimulus was in the memory array) and false alarm rates (proportion of “present” responses in trials in which a test stimulus was not in the memory array) separately for each emotion and WM load condition. Then, *d’* was computed by subtracting the z-score of the false alarm rate from the z-score of the hit rate (Green and Swets, 1966; Macmillan and Creelman, 1991). The WM capacity was estimated using the *k*-iterative (*k_it_*), a modification of Cowan’s *k* suggested by Jackson et al. (2009). To compute *k_it_*, Cowan’s *k* was first calculated separately for each WM load and then averaged across loads. If the average exceeded the *k* at the lowest load, that load was excluded, and the average was recalculated using the remaining loads. This iterative procedure was repeated until the average no longer exceeded the capacity of the lowest remaining load, yielding *k_it_*, which provides a robust estimate of the number of faces that can be maintained in WM while minimizing estimation bias (see Jackson et al., 2009, for details).

For the LTM task on Day 2, we measured the recognition performance for previously encountered facial identities during the WM task on Day 1. Similar to the analysis for the WM task, we computed *d’* separately for each emotional category presented in the WM task: *d’ _happy_* for the pictures presented with a happy expression during the WM task and *d’_angry_* for those with an angry expression. The hit rate was described as the proportion of trials where participants accurately recognized faces shown in the WM task as “old.” Conversely, the false alarm rate was the proportion of trials in which the participants incorrectly identified new faces as “old.” Since these new faces were novel identities not associated with any prior emotion, a single false alarm rate was calculated from these trials and applied to compute *d’* for both happy and angry face conditions.

## 3 Results

Previous studies have reported that social anxiety affects emotional perception (Yang et al., 2012; Yoon et al., 2014) and memory of facial expressions (D’Argembeau et al., 2003b; Kikutani, 2018). To ensure a focus on the effects of emotional valence on memory without the confounding influence of social anxiety traits (e.g., perceptual bias), we excluded seven participants with high social anxiety whose SIAS score was at a clinical cut-off of 37. We also excluded one participant who showed very low performance in the WM task (the proportion of correct responses was below the mean-3SD in both the change-detection and articulatory-suppression tasks). In total, data from 43 participants were analyzed.

**Figure 3:**
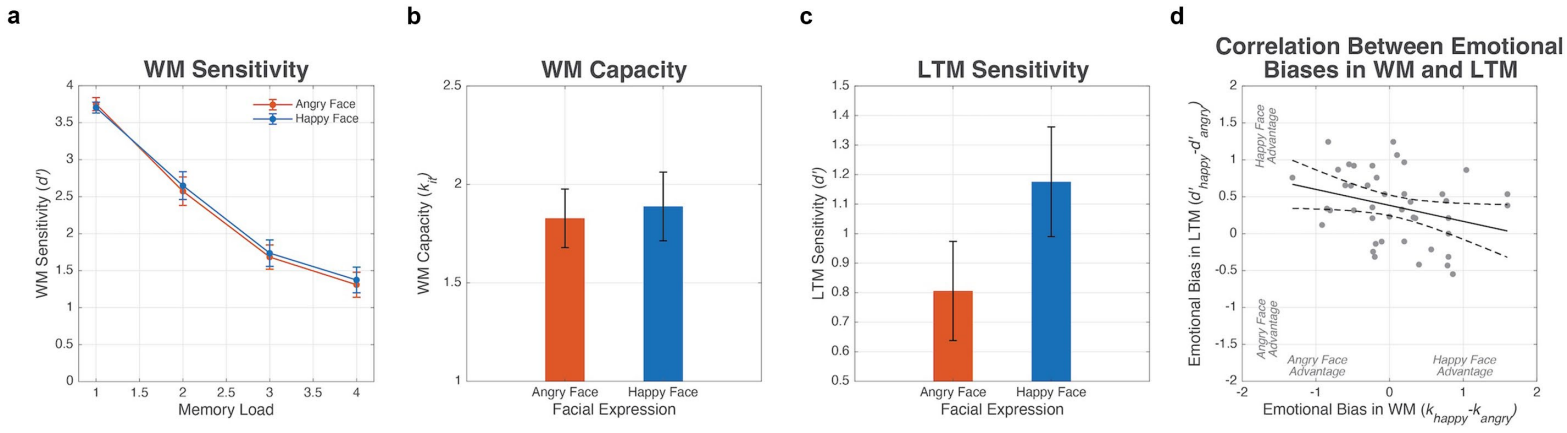
Results from the experiment. **(a)** WM sensitivity, *d’*, as a function of WM load and emotional valence. Error bars represent 95% confidence bounds. **(b)** WM capacity, *k_it_*, for negative and positive emotional expressions. **(c)** LTM sensitivity, *d’*, for different emotional valences. **(d)** Relationship between the happy face advantages in WM and LTM. The solid line represents the regression line, and dashed lines represent 95% confidence bounds.

For the WM task performance, a repeated-measures ANOVA was performed on the *d’* scores, with the facial emotion (angry vs. happy, reflecting the emotion displayed during encoding on Day 1) and WM load (1, 2, 3, and 4 faces) as within-subject independent variables. There was no significant main effect of the facial expression, *F*(1, 42) = 0.48, *p* = 0.492, *η_p_^2^* = 0.01, indicating that memory performance did not differ significantly between angry and happy faces. Additionally, there was a significant main effect of the WM load, *F*(3, 126) = 449.62, *p* < 0.001, *η_p_^2^* = 0.91, demonstrating that WM sensitivity decreased as the number of faces increased in the memory array. Furthermore, the interaction between facial expressions and the WM load was not significant, *F*(3, 126) = 0.34, *p* = 0.795, *η_p_^2^* = 0.01, suggesting that emotional expressions did not interact with the cognitive load (see Figure 3a).

In addition to the repeated-measures ANOVA, we performed a paired *t*-test to compare the WM capacity, *k_it_*, between angry and happy faces. The analysis indicated that the participants did not show significantly different WM capacities for different facial expressions (M = 1.83, SD = 0.50 for angry faces; *M* = 1.89, SD = 0.58 for happy faces), *t*(42) = −0.60, *p* = 0.554, *d* = −0.09 (see Figure 3b). This suggests that the participants’ WM capacity was similar for both emotional expressions, indicating that neither happy nor angry faces provided a distinct advantage in retaining their facial identities during the task. These results suggest that emotional expressions of faces do not have a differential effect on the WM performance, challenging previous findings that angry faces enhance WM compared to happy faces.

For data from the LTM recognition task on Day 2, a paired t-test was performed to compare LTM sensitivity for faces associated with different emotional expressions. There was a statistically significant difference in *d’* between the two emotional categories of the faces encountered during the initial WM task, *t*(42) = −5.17, *p* < 0.001, *d* = −0.79 (see Figure 3c). The results showed that participants recognized faces they had previously seen with happy expressions (*M* = 1.18, SD = 0.62) more accurately than faces associated with anger (*M* = 0.81, SD = 0.56).

To examine the relationship between the emotional effects on WM and LTM, a correlation analysis was conducted between the bias scores of WM and LTM for all the participants. Given that overall memory ability can influence performance across both emotions, we calculated bias scores to isolate the emotion-specific effects within each memory system. Specifically, individuals with generally high memory capacity tend to perform well across both happy and angry expressions, as reflected in a modest positive correlation between WM capacity for the two emotions (*r* = 0.26, *p* = 0.089) and a strong correlation between LTM sensitivity for the two emotions (*r* = 0.69, *p* < 0.001). These results suggest that raw performance measures may be substantially influenced by general memory ability, potentially masking emotion-specific trade-offs. The bias score of WM was calculated as the difference in WM capacity between different emotions (i.e., *bias_wm_* = *k_happy_* − *k_angry_*). Similarly, the bias score of LTM was calculated as the difference in LTM sensitivity between different emotions (i.e., *bias_ltm_* = *d’_happy_* − *d’_angry_*). For both bias scores, a positive score indicated a happy face advantage and a negative score indicated an angry face advantage for each memory system. The results revealed a negative correlation between the two bias scores (*r* = −0.31, *p* = 0.047; see Figure 3d). The happy face advantage in LTM, as shown by the group-level analysis, appeared to be more pronounced among individuals with a negative WM bias, whereas it was less evident in those with a positive WM bias.

To further investigate the relationship between biases in WM and LTM, we conducted *post hoc* exploratory correlation analyses between the sensitivity biases in WM and LTM separately for each WM load. Sensitivity bias for each load was calculated as the difference in sensitivity (*d’*) between happy and angry faces (e.g., *bias_wm_load1_* = *d’_happy_load1_* − *d’_angry_load1_*). The results showed a load-dependent pattern: a significant negative correlation was found only at the highest WM load (load 4: *r* = −0.35, *p* = 0.022), whereas no significant correlations were observed at lower loads (load 1: *r* = 0.09, *p* = 0.581; load 2: *r* = −0.04, *p* = 0.800; load 3: *r* = −0.11, *p* = 0.482). Given that the average WM capacity was approximately 1.8 items, a memory array with four faces exceeds participants’ capacity, suggesting that the performance trade-off between the two memory systems was most pronounced under conditions of high cognitive demand.

## 4 Discussion

### 4.1 Summary of main findings

This study investigated the relationship between the WM and LTM for emotional facial expressions in the same individual. In a change-detection experiment for WM and a recognition test after a prolonged delay for LTM, significant dissociation was observed between the two memory systems. Specifically, the WM capacity did not show any emotional bias during short retention intervals, whereas the LTM performance demonstrated a happy face advantage. Most critically, the happy face bias for LTM was negatively correlated with that for WM, indicating that emotional expressions modulate WM and LTM differently.

### 4.2 WM for emotional faces

Our results revealed no significant differences in the immediate WM between happy and angry faces. This finding contrasts with previous research that reported superior WM for angry faces. Our failure to replicate the angry face advantage in WM can be attributed to methodological details. In our experiment, each memory array included faces with a single emotion, which could have eliminated the competitive advantage of threatening faces. Previous studies often used mixed-emotion arrays in which angry faces competed with happy or neutral faces for WM resources. In contrast, in homogeneous displays, the threat detection advantage for angry faces could be attenuated because no direct competition for attention occurs during encoding.

Nevertheless, the explanation of the memory array composition and attentional competition still does not explain why we failed to replicate the angry face advantage of WM observed in a study using memory arrays with a single emotion. Jackson et al. (2009) found an angry face advantage in a series of studies that used memory arrays with the same emotional display as in our study. One possible explanation for this discrepancy is the characteristics of the participants in the experiment. In our data analyses, seven participants with high social anxiety (SIAS score > 37) were excluded to ensure a focused analysis of the effects of emotional valence without the confounding influence of social anxiety traits. Previous research suggested that anxiety could modulate the perception and attention to threats and influence WM filtering efficiency (Qi et al., 2014). It can be argued that the absence of emotional bias was due to the exclusion of participants with high social anxiety, in whom the angry face advantage in WM was pronounced. To test this possibility, we re-examined the WM capacity for happy versus angry faces, including screened individuals with high social anxiety. The results showed no significant emotional effect on WM, *t*(49) = −0.26, *p* = 0.795, *d* = −0.04, suggesting that the exclusion of socially anxious individuals, at least within the parameters of our experimental design, was not the main reason for our null result.

Another possibility is the subtle differences in the experimental procedures. A closer inspection revealed that the emotional valence in the memory arrays changed in every block in Jackson’s experiment, whereas it changed in a trial-by-trial manner in our experiment. When angry and happy faces were presented in separate blocks, it is plausible that the participants construed the angry face block as a threatening context, thereby enhancing threat-specific preparedness and increasing the angry face advantage. Inconsistent results across studies suggest that the memory advantage for emotional faces in WM is not robust or universally observed. Rather, it may depend on the complex interplay of task parameters, emotional intensity, and individual differences.

### 4.3 LTM for emotional faces and the positivity bias

In contrast to the null results in WM, our LTM findings demonstrated a clear and significant happy face advantage: recognition performance was better for the happy faces initially encountered in the WM task than for the angry faces. Our results replicated a well-established pattern in the emotional memory literature that holds that positive emotional content is remembered more robustly and for extended periods than negative content (D’Argembeau and Van der Linden, 2007; D’Argembeau et al., 2003a; Shimamura et al., 2006). The finding that the happy face advantage emerged only in the delayed LTM test and not in the immediate WM task further underscores the importance of consolidation processes in shaping the valence effect. This temporal delay allows for the selective strengthening of positive memories, a process that does not appear to operate during a brief WM maintenance period.

### 4.4 Trade-off between memory systems

The negative correlation between WM and LTM emotional biases was the most significant finding of our study. While we observed no performance differences in the WM task, analysis of the “bias score” (the difference in performance for happy vs. angry faces) revealed a significant negative correlation between WM bias and LTM bias. This relationship demonstrates that individuals who showed a relatively smaller positivity bias, or even a negativity bias in WM, subsequently exhibited a stronger positive bias toward happy faces in LTM. Conversely, participants with better WM for happy faces showed a reduced happy face advantage in the LTM. The negative direction of this correlation is particularly noteworthy because it directly contradicts the expectations of the traditional multistore memory model, which predicts positive correlations between enhanced WM and subsequent LTM performance (Atkinson and Shiffrin, 1968; Forsberg et al., 2020).

### 4.5 Arousal-based competition theory

The contradictory patterns observed in the WM and LTM systems can be explained by the arousal-biased competition (ABC) theory (Mather and Sutherland, 2011). This theory proposes that emotional arousal modulates the competition between mental representations, enhancing memory for high-priority stimuli and diminishing memory for low-priority stimuli. Angry faces are considered a high-priority because of their immediate adaptive significance as threat signals and their capacity for automatic attentional capture (Burra et al., 2016; Hansen and Hansen, 1988; Jackson et al., 2014). The arousal elicited by threatening faces amplifies this bottom-up perceptual advantage, causing them to win the competition for limited WM resources and leading to enhanced encoding and maintenance of angry face identities in WM (Jackson et al., 2014). Conversely, happy faces may acquire high top-down priority for long-term social affiliation and foster positive future interactions (Tsukiura and Cabeza, 2008). Positive emotions broaden attention, facilitating the elaborative encoding of a wider array of facial features that are crucial for robust identity recognition (Fredrickson, 2001), thereby benefiting LTM consolidation (D’Argembeau and Van der Linden, 2007). Therefore, arousal enhances the consolidation of long-term goal-relevant happy memories that are prioritized for sustained retention. By distinguishing between the types of priority and their influence on different memory stages, the ABC theory could offer a cohesive explanation for these distinct emotional memory effects.

The ABC theory can explain the negative correlation between emotional bias in two different memory systems. Considering that WM and encoding of information into LTM rely on the same limited pool of cognitive resources (Dolcos et al., 2013), the allocation of cognitive resources toward maintaining threatening information in WM may come at the cost of elaborate encoding processes that support robust LTM consolidation. For example, acute, hypervigilant processing of a threat signal (angry face) could be beneficial for immediate defensive reactions; however, this focused attention may result in a subsequent deficit in memory for the same stimulus, especially when compared to a non-threatening, socially rewarding stimulus (happy face). Conversely, relaxed and less effortful processing of a socially rewarding stimulus may free resources for deep encoding and consolidation, leading to an advantage in LTM. Thus, individuals who deploy extensive resources to maintain threatening faces in WM may have fewer resources available for elaborate encoding processes that support robust LTM consolidation. When emotionally demanding materials burden WM resources, subsequent LTM encoding may be compromised because of the competition for shared neural resources. Conversely, individuals who engage in elaborate encoding strategies that benefit from LTM may exhibit a reduced WM capacity for concurrent processing demands.

This interpretation is further supported by the load-specific correlation results, which showed that the negative trade-off in sensitivity biases between WM and LTM was significant only at the highest memory load (load 4). At lower loads, which fall within or near participants’ WM capacity limits, cognitive resources are likely sufficient to accommodate both the active maintenance of emotional stimuli and their encoding into LTM without competition. However, when the load exceeds this capacity (e.g., maintaining four faces in WM), resource competition becomes evident: individuals who prioritize high-priority emotional stimuli in WM tend to show reduced LTM sensitivity to those stimuli, and vice versa. Thus, the compensatory relationship between WM and LTM occurred specifically under high-load conditions. This finding is consistent with the ABC account, which posits that emotional memory performance reflects capacity limitations and competitive resource allocation across processing stages.

The ABC theory and the resource competition explanation provide a compelling framework for reconciling inconsistencies in the broader literature on emotional memory. Our findings indicated that resource constraints have distinct effects on emotional content, with individuals exhibiting trade-offs rather than uniform benefits across memory systems. This suggests that WM and LTM for emotional stimuli are not simply two independent processes, but are part of a continuous, resource-dependent pipeline. An apparent null result in one domain (e.g., our WM findings) may not signify a lack of emotional influence, but rather a state of equilibrium in a resource allocation trade-off that becomes evident only when both systems are measured and their interrelationship is examined.

However, the negative correlation could be explained by factors other than the ABC theory. For instance, the use of the same facial identity set for both the WM test stimuli and the LTM task could result in the negative correlation. This design allowed the WM test probe to serve as a second encoding opportunity, potentially benefiting LTM more in conditions with higher WM error rates (e.g., testing effect). If retrieval failures were more frequent for angry faces in the WM task, participants might scrutinize these probes more carefully, enhancing LTM for angry faces and thereby contributing to the negative correlation between WM and LTM emotional biases. While the account is theoretically plausible, our empirical data suggest it is unlikely to account for our findings. Our results showed that no significant group-level difference in WM sensitivity or capacity between angry and happy faces. This statistical equivalence indicates that participants experienced retrieval failures at similar rates for angry and happy faces, so differential probe re-encoding cannot account for the disproportionate LTM advantage for happy faces. Furthermore, participants received no feedback during the WM task, and each identity appeared repeatedly across trials, making it unlikely that incorrect trials substantially enhanced LTM for any condition.

It should also be noted that, although the ABC theory provides a compelling framework for interpreting our findings, this study did not directly examine the mechanisms proposed by the theory regarding WM and LTM for emotional faces. Specifically, we did not incorporate physiological or subjective measures of emotional arousal. Additionally, we did not directly manipulate the “priority” of the stimuli, which are central constructs of the theory. Therefore, our ABC-based resource competition account remains one of several possible theoretical explanations for our findings. Further investigation is needed to examine the proposed mechanisms and specific predictions directly.

### 4.6 Limitations and future directions

In this study, some individuals showed a memory advantage for angry faces in WM and a strong advantage for happy faces in LTM, whereas others who had a happy face advantage in WM showed a relatively reduced happy face advantage in LTM. Despite these important findings, the current study could not determine the factors that modulated the individual differences in memory bias. Kikutani (2018) found that the happy face advantage in LTM was reduced in individuals with higher social anxiety. However, in our supplementary correlation analyses, which included screened individuals with high social anxiety, the level of social anxiety was not significantly correlated with either WM (*r* = 0.03, *p* = 0.836) or LTM bias (*r* = 0.20, *p* = 0.167). Further research is necessary to understand the reason for the contradictory results from these two studies and to explore potential moderators of individual differences.

In addition to social anxiety, the relatively homogeneous characteristics of our participant group are another limitation of this study. Young and healthy adults participated in our study, which limits the generalizability of our findings to other special populations, such as children, older adults, and individuals with clinical conditions. For example, previous studies reported that the effect of emotional valence on memory varied with age (Gui et al., 2019; Niu et al., 2024). Age-related changes in emotional processing and memory systems may alter the balance between WM and LTM for emotional content. Future studies should replicate this design with a more diverse population to explore the effects of age, cognitive decline, or psychopathology on emotional memory biases and trade-offs.

Additionally, the absence of a neutral face condition in our WM task design makes it difficult to interpret the null difference between happy and angry faces. The null difference in WM performance between happy and angry expressions could reflect either a lack of emotional enhancement in WM compared to neutral faces or an overall enhancement for emotional faces with no difference between the two emotional categories. Future studies that incorporate a separate neutral face condition in the WM task would clarify this interpretational ambiguity.

Another potential limitation is that low-level facial features may differentially influence performance across the two memory tasks. In the WM task, the test probe was physically identical to the memory item, which may have allowed participants to rely in part on low-level visual features (e.g., the orientation or shape of the eyebrows, eyes, or mouth) for recognition, whereas the LTM task required identity recognition across a change in facial expression. Although this design was essential for assessing robust identity consolidation, the difference in the nature of the target stimulus (perceptual matching in WM vs. identity-based recognition in LTM) represents a methodological asymmetry that should be taken into account when interpreting the trade-offs between the two systems.

Finally, our use of only angry and happy faces limits the generalizability of our findings to other basic emotions such as fear, sadness, and disgust. While these represent two highly salient emotional states with distinct adaptive functions, different negative emotions may show distinct patterns of WM and LTM effects based on their specific adaptive functions and neural substrates. Future studies examining multiple emotional expressions simultaneously could provide a more comprehensive picture of whether resource trade-offs occur broadly across an emotional spectrum.

### 4.7 Conclusion

By measuring the emotional memory for facial expressions in WM and LTM systems within the same individual, we demonstrated apparent dissociation, with an emphasis on a compensatory processing trade-off between the systems. Our findings provided evidence that the cognitive system manages immediate and delayed memories of emotional faces in a complementary fashion. Understanding these trade-offs is a crucial step toward developing more nuanced models of how emotion influences human memory, with a focus on both the temporal dynamics of memory and the valence-specific effects of emotional stimuli.

## Data Availability

The datasets presented in this study can be found in online repositories. The names of the repository/repositories and accession number(s) can be found at: https://osf.io/u5pyz/.
